# Essential role of local antibody distribution in mediating bone-resorbing effects

**DOI:** 10.1038/s41598-024-56192-1

**Published:** 2024-03-07

**Authors:** Merja Nurkkala-Karlsson, Marie K. Lagerquist, Priti Gupta, Claes Ohlsson, Dan Mellström, Cecilia Engdahl

**Affiliations:** 1 Department of Rheumatology and Inflammation Research, Institute of Medicine, Sahlgrenska Academy, University of Gothenburg, Gothenburg, Sweden; 2 SciLifeLab, University of Gothenburg, Box 413, 405 30 Gothenburg, Sweden; 3 Sahlgrenska Osteoporosis Centre, Centre for Bone and Arthritis Research, Department of Internal Medicine and Clinical Nutrition, Institute of Medicine, Sahlgrenska Academy, University of Gothenburg, Gothenburg, Sweden

**Keywords:** Osteoimmunology, Rheumatic diseases, Bone, Osteoimmunology, Humoral immunity, Antibodies

## Abstract

The link between antibodies and bone mass is debated. Activated IgG, which interacts directly with Fc gamma receptors, stimulates osteoclastogenesis in vitro, and local injection in immune-activated mice leads to bone loss. Multiple myeloma patients with high serum IgG levels have induced osteoclast activation and display bone loss. In addition, bone loss has been linked to serum autoantibodies in autoimmune diseases, including anti-citrullinated protein antibodies (ACPA) in individuals with rheumatoid arthritis (RA). Whether serum IgG or autoantibodies regulate bone mass under healthy conditions is poorly studied. In elderly men, neither serum levels of polyclonal IgG nor autoantibody were associated with areal bone mineral density in the MrOS Sweden study. Repetitive systemic injections of high-dose polyclonal IgG complexes in mice did not exert any discernible impact on bone mineral density. However, repetitive local intra-articular injection of the same IgG complexes led to a localized reduction of trabecular bone density. These results indicate antibodies may only impact bone density when close to the bone, such as within the synovial joint.

## Introduction

The immune system influences bone homeostasis, and inflammation often leads to osteoporosis with decreased bone strength and increased risk of fracture^1^. A balance between bone-forming osteoblasts and bone-resorbing osteoclasts intricately regulates bone health. Osteoclast formation is driven by soluble mediators such as macrophage colony-stimulating factor (M-CSF) and the receptor activator of NF-κB ligand (RANKL). Osteoblasts are one of the major producers of M-CSF and RANKL, but local immune cells can also produce these regulators. Furthermore, immune cells can potentially enhance osteoclastogenesis by producing pro-inflammatory cytokines. Compelling evidence presented by us and others shows that antibodies, both activated IgG and autoantibodies, can impact osteoclasts, consequently leading to bone loss.

IgG is the most common form of antibody found in serum. Upon activation, IgG interacts with Fc-gamma receptors (FcγRs) and can regulate the function of all hematopoietic cells, including osteoclasts^2–4^. We and others have shown that activated IgG enhance RANKL-mediated in vitro osteoclastogenesis in human and murine cell models and induce local immune-mediated bone loss in vivo in mice^2–8^. Patients with myeloma, a hematological malignancy in plasma cells with high serum IgG levels, have induced osteoclast function^9^. In addition, we have described that muMT mice, lacking IgG, IgM, the majority of IgA and B cells, have increased trabecular bone mineral density^10^.

Bone loss is a pathological hallmark of several autoimmune diseases, for instance, rheumatoid arthritis (RA) and systemic lupus erythematosus (SLE). Besides pro-inflammatory cytokines, bone loss is positively associated with autoantibodies, including anti-citrullinated protein antibodies (ACPA) in RA patients^11–13^ and anti-nucleated antibodies (ANA) such as anti-double strain DNA (anti-dsDNA) antibody-positive in SLE patients^14,15^. ACPA can be detected in serum years before the clinical manifestation of RA, serving as a highly specific predictive marker^16,17^. ACPA-positive individuals, even without joint pain or inflammation, exhibit decreased bone mass before disease onset^18,19^. In addition to RA, senescence is associated with serum levels of ACPA in a large population-based cohort of healthy individuals^20^. In RA patients, the inflammation is positioned, and therefore, the antibodies are present within affected joints, and osteoclasts are one of the cell types presenting citrullinated proteins, the antigen for ACPA^21,22^. Using a murine model, we recently described a direct link between ACPA and arthritis-mediated bone loss^23^. ANA positivity has also shown a link to bone loss in murine models^24,25^.

While the connection between autoantibodies, particularly ACPA, and bone loss in autoimmunity is well established, the direct relationship in healthy conditions is less clear. This study explores the potential association between serum levels of polyclonal IgG and autoantibodies with bone mass in the well-established MrOS Gothenburg cohort in elderly men. Additionally, we investigate if local or systemic injections of polyclonal IgG complexes affect bone loss in mice.

## Results

### There is no association between serum levels of polyclonal IgG or autoantibodies and bone mineral density in elderly men

To address whether serum levels of polyclonal IgG and autoantibodies such as ACPA and anti-dsDNA antibodies are associated with human bone mass, we assessed the serum of the follow-up study to MrOS Sweden. In the cohort of elderly men, including 545 individuals, the median level of ACPA was 3.39 [2.1–5.7] U/ml. Twelve of the men were considered ACPA-positive, according to the disease criteria for RA. No significant difference in aBMD was observed between ACPA-positive (n = 12) and ACPA-negative (n = 533) participants (*femur neck BMD*, beta = -0.085, *P* = 0.75, *lumbar spine BMD*, beta = − 0.394, *P* = 0.15, beta is expressed as SD change in BMD in a linear regression model adjusted for age, height, and weight). In 481 elderly men, the median level of anti-dsDNA antibodies was 4.81 [3.8–6.5] U/ml, and none of the men were considered anti-dsDNA positive according to the disease criteria for SLE. The median of polyclonal IgG in the 528 elderly men was 1012 [500–1313] mg/dL. Neither serum polyclonal IgG nor autoantibody levels were associated with areal bone mineral density in elderly men (Table 1).

**Table 1:** No associations for serum polyclonal IgG or ACPA with femoral neck areal bone mineral density (aBMD) or lumbar spine aBMD in elderly men.

	Femur neck aBMD				Lumbar spine aBMD			
	Beta	SE	P	N	Beta	SE	P	N
log IgG adjusted	-0.023	0.041	0.580	523	0.021	0.041	0.604	528
log ACPA adjusted	0.023	0.039	0.553	540	0.003	0.039	0.932	545
log anti-dsDNA adjusted	-0.074	0.042	0.079	476	0.067	0.041	0.105	481

Serum levels of IgG, anti-citrullinated protein antibody (ACPA), and anti-double strain DNA (anti-dsDNA) were measured using commercially available ELISA kits. Femur neck aBMD and lumbar spine aBMD were measured with dual-energy-x-ray absorptiometry (DXA). Associations between serum levels and bone mineral density were determined using linear regression models adjusted for age, height, and weight. Betas are expressed as standard deviation (SD) change in bone mineral density per SD increase in log-transformed serum levels. SE = standard error.

### Systemic administration of IgG complexes does not affect the bone

**Figure 1:**
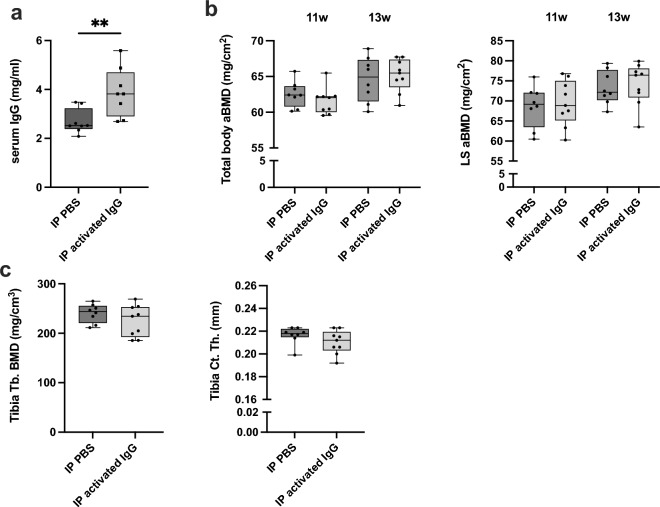
Effects of systemic injections of activated IgG complexes in mice. 11-week-old female mice were given intraperitoneal (IP) injections with activated polyclonal IgG complexes (100 mg/kg) or PBS. IP challenges were repeated after one week, and after an additional week, the mice were terminated. PBS *n* = 8, IgG* n* = 10. Student’s t-test was used to assess differences between mice challenged with activated IgG or PBS. *p < 0.05 (**a**) IgG serum levels were measured using commercially available ELISA kits. All individual data, median, interquartile range, and max and min values are shown in boxplots. (**b**) Dual-energy-x-ray absorptiometry (DXA) analyses were used to assess the total body and lumbar spine (L3-L6) areal bone mineral density (aBMD) over time. DXA analyses were performed before the first injection (11 weeks) and at termination (13 weeks). (**c**) Trabecular (Tb.) bone mineral density (BMD) and cortical (Ct.) thickness (Th.) were investigated with peripheral quantitative computed tomography (pQCT). All individual data, median, interquartile range, and max and min values are shown in boxplots.

The impact of IgG complexes on bone was investigated in mice through repeated systemic injections. Systemic IgG complex injections resulted in 1.4 times higher serum IgG levels (Fig. 1a). This treatment did not affect total body, liver, or spleen weight (Supplementary Table 1). The skeleton was analyzed with DXA, and IgG complexes did not promote alterations in either total body or lumbar spine aBMD (Fig. 1b). There were neither any alterations in tibia trabecular BMD or cortical thickness after detailed investigation using pQCT analysis (Fig. 1c).

### Local injection with IgG reduces trabecular bone

The local impact of IgG on bone was investigated in mice with repeated intra-articular injections of IgG complexes. Intra-articular IgG complex challenge did not change total body, liver, or spleen weights compared to naïve control mice (Supplementary Table 2), and no effects were seen on serum IgG levels or total body aBMD compared to the naïve control mice (Supplementary Fig. 3). When comparing the IgG complex-challenged knee to the PBS-challenged side, no alteration of inflammation was detected, determined by swelling over the knee and histological assessment of inflammation within the joint (Supplementary Fig. 2).

**Figure 2:**
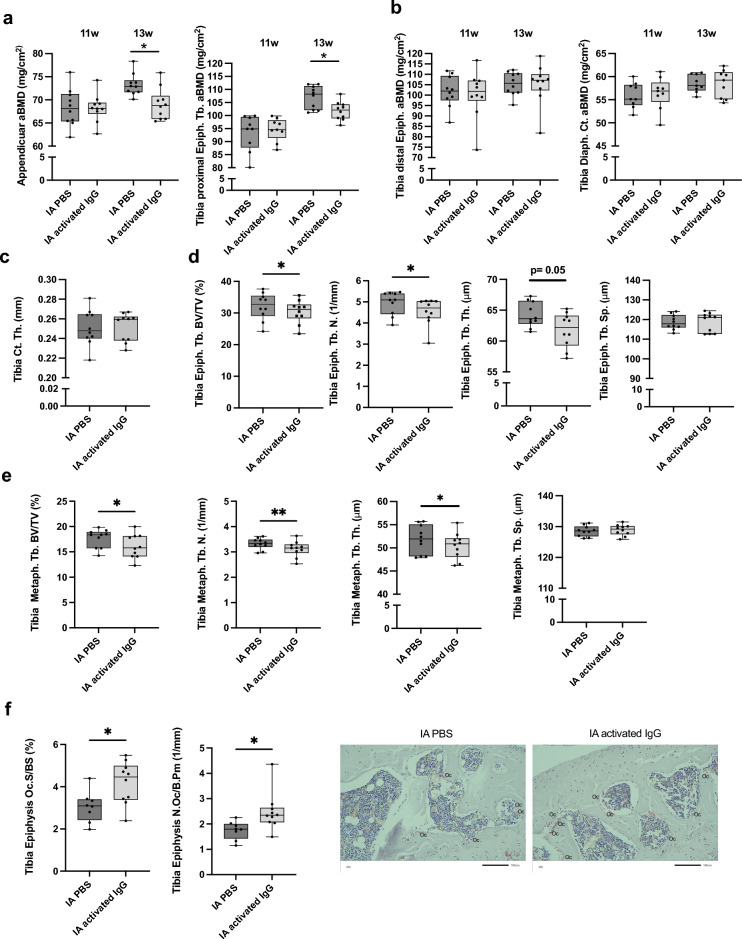
Effects on the bone of local injections of activated IgG complexes in mice. 11-week-old female mice were given an intra-articular (IA) challenge with activated IgG complexes in one knee and PBS in the contralateral knee (internal control). IA challenges were repeated after one week, and after one additional week, the mice were terminated. (*n* = 10). A two-sided paired Student’s t-test was used for internal comparisons between the differently treated knees in the mice. *p < 0.05. **p < 0.01. (**a**) Dual-energy-x-ray absorptiometry (DXA) analyses were used to assess appendicular areal bone mineral density (aBMD) (tibia and femur) and the proximal epiphyseal (Epiph.) trabecular (Tb.) bone of the tibia (**b**) DXA analyses of diaphyseal (Diaph.) part of tibia and the distal Epiph. Tb. aBMD part of the tibia over time. DXA analyses were performed before the first injection (11 weeks) and at termination (13 weeks). Micro-CT was used to assess the tibia in more detail. (**c**) Cortical (Ct.) thickness (Th.) in the mid-diaphyseal region of the tibia. (**d**) Trabecular investigation in the top part of the tibia, Epiph. Tb. Bone volume/tissue volume (BV/TV), Tb. Number (N), Tb. Thickness (Th.) and Tb. Separation (Sp). (**e)** Metaphyseal (Metaph.) part of the tibia, below the growth plate, Tb. BV/TV, Tb. N., Tb. Th., and Tb. Sp. (**f**) Data show osteoclast in the proximal epiphyseal part of the tibia, osteoclast surface per bone surface (Oc.S/BS), and the number of osteoclasts per bone perimeter (N.Oc/B.Pm). Pictures representing each group, osteoclasts (Oc) are marked out. All individual data, median, interquartile range, and max and min values are shown in boxplots.

Interestingly, we found a reduction in total appendicular aBMD (including both femur and tibia) and aBMD of the trabecular bone in the proximal epiphyseal part of the tibia close to the injected knee (Fig. 2a). No alteration was found in diaphyseal (cortical) aBMD nor the distal epiphyseal part close to the foot after intra-articular IgG complex injection compared with the PBS-injected internal control knee (Fig. 2b). In the tibia, high-resolution micro-CT analyses demonstrated that intra-articular injections with IgG complexes did not affect cortical thickness in the diaphyseal bone (Fig. 2c). Both epiphyseal, and metaphyseal trabecular bone volume fractions (BV/TV) in the proximal part of the tibia were reduced (Fig. 2d,e). Detailed analysis of the trabecular bone showed reduced trabecular number and reduced trabecular thickness in the metaphyseal bone. The epiphyseal trabecular number was reduced and a tendency to reduction of trabcecular thickness. We did not observe any alteration in trabecular separation. Finally, we observed an increase in osteoclast surface per bone surface and the number of osteoclasts per bone perimeter in the trabecular epiphyseal part of the tibia (Fig. 2f).

## Discussion

This study investigated the association between levels of polyclonal IgG and autoantibodies to bone mass using a well-established cohort of elderly men. We also investigated if local or systemic challenges with high doses of polyclonal IgG complexes affect bone mass in mice. It is well-established that autoantibodies and deposition of activated IgGs in the joint stimulate bone loss in patients with RA^23,26,27^. However, there is no association between levels of serum polyclonal IgG and autoantibodies, ACPA and anti-dsDNA antibodies, to bone mineral density in a cohort of elderly men. Furthermore, systemic challenges with IgG complexes do not alter bone mass in mice. However, in line with previous findings, local challenges with activated IgG complexes in the joint lead to adjacent trabecular bone reduction.

In our study, 2.2% of the elderly men were considered ACPA-positive. In a large population-based study (Lifeline), 1% of healthy individuals were considered ACPA positive^20^. However, in the Lifeline study, ACPA-positivity was more frequently present in the elderly. Thus, the high average age in the MrOS cohort (80 years) may contribute to the higher frequency of ACPA positivity compared to the Lifeline study. In several studies, serum ACPA has been linked to bone loss in pre-RA and established RA patients^11,12,18,19^. This was not the case in our study of healthy elderly men, which showed no association between serum ACPA levels and femur neck or lumbar spine areal bone mineral density. Furthermore, the aBMD did not differ between ACPA-positive and ACPA-negative participants. A possible explanation for this discrepancy is that bone mass alteration depends on immune activation. RA patients have clinical or at least subclinical activated immune systems, whereas elderly men have only limited immune activation. This may limit the capability of ACPA to induce bone loss. Another possible explanation is that in RA patients, it is well known that ACPA is present in the joint, and osteoclasts express citrullinated vimentin, the antigen for ACPA. Whereas, in the MrOS cohort, the location of the antigen for ACPA is not known and citrullinated protein could be expressed in a variety of tissues.

None of the elderly men were considered anti-dsDNA antibody positive. To our knowledge, anti-dsDNA antibodies have not previously been measured in healthy individuals. But ANA, which includes anti-dsDNA antibodies, are present healthy individuals, in a proportion ranging from 2.5% to 17%^28,29^, with a higher prevalence of positive individuals among females compared to males^29^. Our material did not detect any association between serum levels of anti-dsDNA antibodies and areal bone mineral density in the femoral neck or lumbar spine. This aligns with a previous study by Iseme et al., where neither ANA- nor ACPA-positive individuals showed a significant association with bone mass in individuals without autoimmune diseases^29^.

The mean serum polyclonal IgG levels in MrOS are comparable to a previous study in elderly men^30^. Contrary to our hypothesis, the serum polyclonal IgG was not associated with areal bone mineral density in the MrOS Gothenburg study. In line with this, we systemically injected mice with high doses of IgG complexes. An increased serum level of IgG was detected in serum indicating a sufficiently injected. However, we did not see any alteration of the bone mineral density neither in the tibia nor total body. Serum IgG has not previously been compared to bone mass. However, patients with multiple myeloma, a hematological malignancy with high serum IgG levels, are prone to develop osteoporosis^31^. Increased levels of monoclonal IgG from multiple myeloma patients, especially de-glycosylated IgG, lead to elevated activation of osteoclasts in vitro^32^. Confirming these results, we, and others, have shown that activated polyclonal IgG or IgG can stimulate osteoclastogenesis in vitro and that Ig or B cells may alter the trabecular bone mass^3,5,7,8^. To reconcile our data demonstrating that serum IgG does not alter bone mass, no one has previously shown that polyclonal IgG without proximity to the bone influences bone mass. The effect mediated by IgG, as well as autoantibodies, might be dependent on the location rather than dependent on the presence. We speculate that the polyclonal IgG or autoantibody concentration that reaches the bone may be too low in the MrOS material. The same concept also applies to systemic injection of IgG complexes in mice.

Intra-articular injections with high-dose IgG complexes reduce trabecular bone mass and induce osteoclasts in the proximal epiphyseal region of the tibia adjacent to the injection site in mice. This is in accordance with earlier observation of how intra-articular injection of IgG complexes affects the trabecular bone^3,5,10^. These earlier observations were performed in immune-induced conditions, including TNF stimulus^3^, prior immunization with Freud’s complete adjuvant^5^, or in muMT mice, which lack B cells and Ig^10^. In this study, we used wild-type naive mice with no immune stimulation, which offers the possibility to study the effect of local IgG stimulation on bone loss in local circumstances under healthy conditions. An explanation for the observed reduction in bone mass might be that activated IgG directly can interacts with FcγRs on osteoclasts in naïve conditions, thereby contributing to bone loss. One interesting finding was that we could observe a reduction of the trabecular bone of the tibia adjacent to the injection site but not in the cortical bone. Cortical and trabecular bones share the same cell types but have considerable structural differences. This and the proximity to the site of injections may explain the different effects between the two bone compartments.

In our study, limitations arose due to constraints in sample size, particularly affecting our ability to conduct subgroup analyses. Our focus was to investigate the relationship between the levels of serum polyclonal IgG and autoantibody to bone density in healthy elderly men rather than performing subgroup categorizations. Additionally, uncertainties emerged regarding the persistence of IgG complexes upon entering circulation post-intraperitoneal injection. We detect elevated values of IgG in serum after the intraperitoneal injection. However, we can’t verify that the IgG is still active in a complex and can immediately affect the bone via FcγRs.

Our study challenges the notion that serum antibodies are important for bone loss. The results demonstrate that serum polyclonal IgG and autoantibodies are not associated with bone mass in conditions without immune induction in the cohort of MrOS. In addition, the systemic challenge of mice with IgG complexes did not affect bone mass. However, local challenges with IgG complexes decreased local trabecular bone mass and increased osteoclasts. This indicates that activated IgG could stimulate healthy trabecular bone loss, but only when it’s situated close to the bone. In RA or pre-RA patients, the antibodies are most likely present within the joint near the bone, which could explain why bone loss is induced in these patients.

## Methods

### Human study population

Serum samples, lumbar spine, and femur neck areal bone mineral density (BMD) measurements were available for 546 elderly men in the MrOS Sweden, Gothenburg cohort (median age 80 years). These men were part of the Swedish MrOS (Osteoporotic Fracture in Men) cohort study, a multinational epidemiological study of risk factors for osteoporosis and fractures in elderly men^33^.

#### Serum analyses

Using the ELISA method, we analyzed levels of total polyclonal IgG, ACPA, and anti-dsDNA antibodies in serum levels. Commercially available ELISA kits (Orgentec. Mainz. Germany) were utilized to investigate autoantibody levels. The serum dilution in the sample buffer was adjusted to enhance the detection of lower antibody levels (1:10, as opposed to the standard clinical practice of 1:100) after discussion with Dr Holger Bang at Orgentec. The ACPA ELISA has a lower quantification (LOQ) limit of 1 unit/ml, with an upper limit of 1000 units/ml. In RA diagnostics, samples exceeding 20 units/ml in 1:100 dilution are considered positive, and this was transferred to 200 units/ml in our 1:10 dilution. One sample failed the assay due to the absence of serum in the well, one sample with antibody levels below the LOQ was adjusted to 1 unit/ml, and another sample with levels exceeding the upper limitation was adjusted to 1000 units/ml. The anti-dsDNA antibodies assays has a LOQ of 1 unit/ml and an upper limit of 200 units/ml. In SLE diagnostics, samples above 20 units/ml in 1:100 dilution are considered positive, and this is transferred to 200 units/ml in the 1:10 dilution. 65 samples failed the assay due to handling failure with one plate. However, all the remaining samples were within the 1–200 units/ml detection range.

The levels of total polyclonal IgG were evaluated according to protocol using a commercial Human IgG ELISA Quantitation Set (Bethyl Laboratories, Nordic Biosite, Taby, Sweden), with a dilution of 1:60 000. The LOQ was 6.9 ng/ml, and the upper limitation was 500 ng/ml. Unfortunately, 18 samples failed in the assay due to technical difficulties affecting the reading of a part of the ELISA plate. Additionally, three samples had values below LOQ and were adjusted to 6.9 ng/ml, while 18 samples exceeded the upper limit and were adjusted to 500 ng/ml. Since the aim was to determine the association between serum levels of antibodies and bone mass, all individuals with measurable antibody levels were included even though the levels were below the conventional disease criteria levels. Log transformation was performed as serum levels of polyclonal IgG, and ACPA were not normally distributed.

#### X-ray analyses

Lumbar spine (n = 546) and femoral neck (n = 541) areal BMD (aBMD) was determined in the elderly men with serum analyses available using Hologic QDR 4500/A-Delphi DXA (Hologic, Waltham, MA, USA)^33^.

### Animal study

C57BL/6 J mice (Taconic Bioscience, Ejby, Denmark) were bred and housed at the Sahlgrenska Academy, Laboratory of Experimental Biomedicine in Gothenburg, Sweden, and fed phytoestrogen-free pellet diet (Harlan 2016) ad libitum*.*

#### Bone loss induction by polyclonal IgG complexes

Polyclonal IgG was isolated from pooled serum of intact C57BL/6 J mice of different ages and sexes using a protein G spin column (GE Healthcare, Sigma-Aldrich, Sollentuna, Sweden) according to the manufacturer’s instructions. IgG and protein concentrations were determined using a Mouse IgG ELISA Quantitation set (Bethyl laboratories, Nordic Biosite) and detergent-compatible protein assay (Bio-Rad Laboratories AB, Solna, Sweden), respectively, according to the manufacturer’s instructions. Polyclonal IgG was activated to form complexes by heat-aggregation at 63 °C for 30 min. The IgG complexes can interact with FcγRs without antigen activation^3,6^. The sample size was calculated with the G*power software using data from a previous study in which polyclonal IgG complexes were injected in systemically immune-induced mice to induce trabecular bone loss^5^. With an alpha value of 0.05, a power of 80%, and a calculated Cohen’s d effect size of 1.66, our study would require a sample size of 6 per group to attain significance. The effect of systemic administration of polyclonal IgG complexes (100 mg/kg) or PBS was assessed by intra-peritoneal (IP) injections in 11-week-old female mice (PBS n = 8, IgG n = 10). The local effect of polyclonal IgG complexes was assessed by an intra-articular (IA) injection (7.5 mg/kg) in one knee. The contralateral knee was injected with PBS, an internal control (n = 10). The IA and IP injections were repeated after one week, and after an additional week, the mice were terminated. Swelling over the knees was determined using a caliper following IA injection. In the IA-injected group, an extra set of naïve mice was used as a control for systemic effect (n = 4).

#### Murine serum analyses

IgG serum levels were measured using a commercially available mouse IgG ELISA kit (Bethyl Laboratories, Nordic Biosite).

#### X-ray analyses

Dual-energy-x-ray absorptiometry (DXA) analyses were performed to assess aBMD in mice using Faxitron UltraFocus^DXA^ (Faxitron Bioptics. Tuscon. AZ. USA) of 40 kV and 0.28 mA for 2.53 s with the spatial resolution of 24 µm using 2X geometric magnification as previously described^5^. Total body aBMD and lumbar spine aBMD (L3-L6) were investigated before and two weeks after systemic injections. Total body aBMD, appendicular (tibia and femur) aBMD, proximal tibia epiphyseal aBMD (trabecular part of tibia close to knee), distal tibia epiphyseal aBMD (trabecular part of tibia close to the foot) and diaphyseal aBMD (cortical bone) were assessed before and two weeks after intra-articular injections.

After systemic injections with IgG complexes, mice’s tibial trabecular and cortical bone were analyzed with Stratec peripheral Quantitative Computed Tomography (pQCT) XCT Research M (software version 5.4, Norland) at the resolution of 70 µm. The trabecular BMD region was defined in the metaphysis at 0.4 mm from the proximal growth plate in the distal direction by setting an inner area to 45% of the total cross-sectional area. Cortical thickness was determined by analyzing scans of the mid-diaphyseal region of the tibia at a distance corresponding to 36% of the total length from the growth plate.

After intraarticular injection of IgG-complexes, mice’s tibial trabecular and cortical bone were analyzed with high-resolution microcomputed tomography (micro-CT, Skyscan 1172 scanner, Bruker Micro-CT, Belgium). The trabecular bone of the tibia was investigated within a conforming volume of interest (cortical bone excluded) distally (metaphysis) and proximally (epiphysis) of the proximal growth plate. The cortical micro-CT measurements in the tibia were performed on the mid-diaphyseal region of the tibia.

#### Histological examination

Hind limbs were fixed in 4% formaldehyde, decalcified in 10% EDTA, paraffin-embedded, and cut into 4 µm thick sagittal sections (sectioning was performed at Histocenter, Mölndal, Sweden). Knees injected IA with IgG complexes were stained using hematoxylin and eosin. Synovitis, bone erosion, and cartilage destruction were graded using a blunt 3-grade system by an examiner (CE) blinded to the interventions, as described by Liphardt et al.^34^. To quantify osteoclast numbers and osteoclast surface attached to the bone, knee sections after IA injections with IgG complexes were stained with tartrate-resistant acid phosphatase (TRAP) buffer including Sodium Acetate Solution, Acetic acid Solution, Acetate Buffer, Sodium Tartrate as well as Naphthol AS-MX (Sigma-Aldrich, Sollentuna, Sweden). Osteoclasts were defined as TRAP-positive cells on the bone in the epiphyseal part of the tibia proximal to the growth plate. All analyses were performed using a microscope (Nikon, Tokyo, Japan) and the image analysis system Osteomeasure (OsteoMetrics, Decatur, GA, USA).

#### Statistics

Analyses were performed with GraphPad Prism software (Graph Pad Software Inc., La Jolla, CA, USA) or SPSS software 21.0 (IBM, Armonk, NY, USA). Normal distribution was confirmed in all animal studies with the Shapiro–Wilk normality test. The statistical difference between two independent animal groups was calculated using a two-sided unpaired Student’s t-test. A two-sided paired Student’s t-test was used when internal comparisons were performed between the different knees in the mice. The non-parametric Mann–Whitney test was used for ordinal scale comparisons of the histological scoring after IA injection.

Tables and DXA figures showing data over time are given as mean ± SEM. In other figures, all data are plotted as boxplots with median, interquartile range, and max and min values. *P* < 0.05 was considered significant.

#### Ethics

The MrOS cohort study was approved by the ethical review board at the Sahlgrenska University Hospital and University of Gothenburg, and all participants signed informed consent. All sample handling and procedures were performed and reported according to relevant guidelines and regulation approved at the Sahlgrenska University Hospital and University of Gothenburg.

The animal ethics committee approved the animal study in the Gothenburg region (1–2017). All experiment procedures and animal handling were performed and reported according to relevant guidelines and regulations, including ARRIVE guidelines.

### Supplementary Information

Supplementary Tables.

Supplementary Information 1.

Supplementary Figures.

## Data Availability

We will share data supporting this study’s findings from the animal studies, which are available in the figshare repository,  https://figshare.com/s/c0787298b780a9e82b82.
