# Supplementary material for: Essential role of local antibody distribution in mediating bone-resorbing effects
Source: Sci Rep. 2024 Mar 7;14:5684. doi: 10.1038/s41598-024-56192-1 (PMC10920837; doi:10.1038/s41598-024-56192-1)
Supplement: Supplementary file 1 — Supplementary Tables. [file 41598_2024_56192_MOESM1_ESM.docx]

**Supplementary Table 1**

|  | **PBS** | **IgG complexes** |
| --- | --- | --- |
| ***Total body (g)*** | 21.06 ± 0.37 | 20.91 ± 0.30 |
| ***Liver (mg)*** | 850.1 ± 29.6 | 907.2 ± 25.5 |
| ***Spleen (mg)*** | 89.3 ± 7.8 | 92.5 ± 6.3 |

**Supplementary Table 1 Intraperitoneal (IP) injections with activated IgG complexes do not affect the weight of the body, liver, or spleen compared to PBS controls.**

11-week-old female mice were given IP injections with activated polyclonal IgG complexes (100mg/kg) or PBS repeatably at weeks 11 and 12. The mice were terminated one week later, and total body, liver, and spleen weights were measured. PBS *n*=8, IgG *n*=10. Student’s t-test was used to assess statistical differences between mice challenged with IgG complexes or PBS. Data are shown as average ±SEM.

|  | **Naïve control** | **IA injections IgG complexes** |
| --- | --- | --- |
| ***Total body (g)*** | 19.77 ± 0.09 | 20.58 ± 0.39 |
| ***Liver (mg)*** | 884.9 ± 18.5 | 904.4 ± 21.4 |
| ***Spleen (mg)*** | 66.4 ± 3.1 | 76.2 ± 3.9 |

**Supplementary Table 2**

**Supplementary Table 2 Intra-articular (IA) injections with activated IgG complexes do not affect the body weight, liver, or spleen weights compared to naïve controls.**

11-week-old female mice were given intra-articular (IA) injections with activated IgG in one knee repeatably at weeks 11 and 12. One week later, the mice were terminated, and total body, liver, and spleen weights were measured (*n*=10). Naïve mice were used to control for systemic effects (*n*=4). A two-sided unpaired Student’s t-test assessed statistical differences between mice challenged with IA-injected activated IgG-complexes and naïve mice. Data are shown as mean ±SEM.
