# Supplementary material for: Essential role of local antibody distribution in mediating bone-resorbing effects
Source: Sci Rep. 2024 Mar 7;14:5684. doi: 10.1038/s41598-024-56192-1 (PMC10920837; doi:10.1038/s41598-024-56192-1)
Supplement: Supplementary file 2 — Supplementary Information 1. [file 41598_2024_56192_MOESM2_ESM.docx]

**Supplementary Figure 1: Repeated local injections with activated IgG complexes do not give systemic effects.**

11-week-old female mice were repeatedly given intra-articular (IA) injections with activated IgG complexes in one knee and PBS in the contralateral knee (internal control) at weeks 11 and 12 to assess the local effects on bone. One week later, the mice were terminated. (n=10). Naïve mice were used to control for systemic effects (n=4). The statistical difference was calculated using a two-sided unpaired Student’s t-test. **a)** IA injections with activated IgG complexes do not give higher levels of serum IgG compared to naïve controls. **b)** Total body areal bone mineral density (aBMD) was unaffected by IA injections with activated IgG-complexes compared to naïve mice. Dual-energy-x-ray absorptiometry (DXA) analyses were performed before the first injection (11 weeks) and at termination (13 weeks). All individual data, median, interquartile range, and max and min values are shown in boxplots.

**Supplementary Figure 2: No signs of inflammation after local injection with activated IgG**

11-week-old female mice were given intra-articular (IA) injections with IgG complexes in one knee and PBS in the contralateral knee (internal control). IA injections were repeated after one week, and after one additional week, the mice were terminated. (n=10). Inflammation was assessed by histological examination. Synovitis, bone erosion, and cartilage destruction were graded. All individual data are shown with median and SEM.
