# Supplementary figures and images for: Essential role of local antibody distribution in mediating bone-resorbing effects
Source: Sci Rep. 2024 Mar 7;14:5684. doi: 10.1038/s41598-024-56192-1 (PMC10920837; doi:10.1038/s41598-024-56192-1)

Supplementary Fig 1

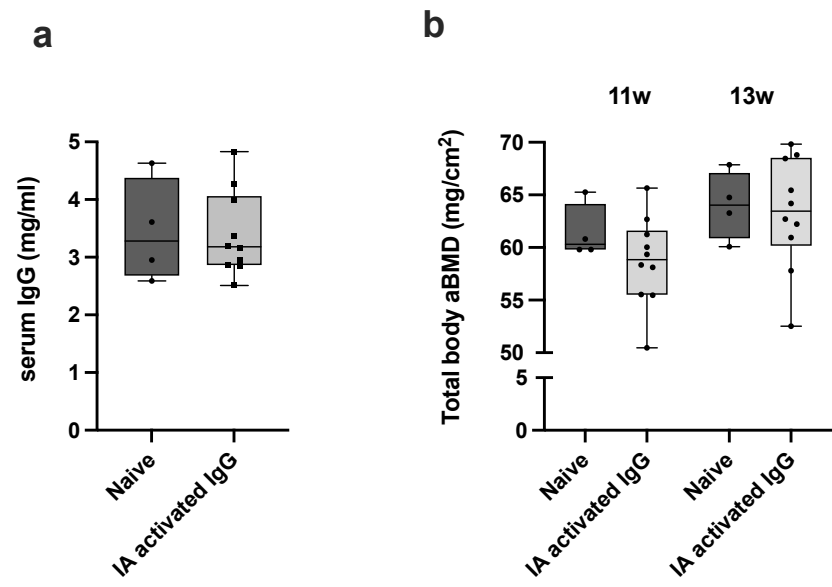

Supplementary Fig 2

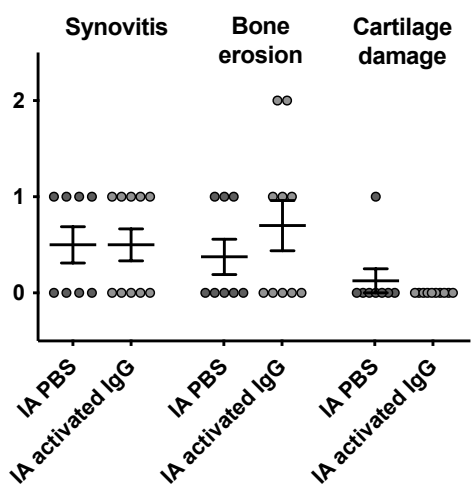

Supplement: Supplementary file 3 — Supplementary Figures. [file 41598_2024_56192_MOESM3_ESM.pdf]
